# Machine learning identifies potential accessory resistance-associated mutations in HIV-1 integrase

**DOI:** 10.1186/s12864-026-13243-3

**Published:** 2026-08-03

**Authors:** Alfred Ssekagiri, Deogratius Ssemwanga, David Patrick Kateete, Daudi Jjingo

**Affiliations:** 1 Department of General Virology, Uganda Virus Research Institute (UVRI), Entebbe, Uganda; 2 Medical Research Council (MRC)/UVRI and London School of Hygiene and Tropical Medicine (LSHTM) Uganda Research Unit, Entebbe, Uganda; 3 Department of Immunology and Molecular Biology, College of Health Sciences, Makerere University, Kampala, Uganda; 4 African Center of Excellence in Bioinformatics and Data Intensive Sciences, Infectious Diseases Institute, Makerere University, Kampala, Uganda; 5 Department of Computer Science, College of Computing and Information Sciences, Makerere University, Kampala, Uganda

**Keywords:** HIV-1 integrase, Drug resistance mutations, Machine learning, Antiretroviral therapy, Epistatic interactions

## Abstract

**Background:**

Although integrase strand transfer inhibitors (INSTIs) have a high genetic barrier to resistance, cases of virological failure continue to emerge, sometimes in the absence of major resistance-associated mutations. Conventional genotypic and phenotypic resistance testing is costly and time-intensive, especially in resource-limited settings, and is inherently limited in detecting novel resistance pathways in any setting. Machine learning offers a scalable approach to uncover previously unrecognized resistance-associated patterns in HIV-1 genomic data.

**Results:**

We analyzed 41,247 publicly available HIV-1 integrase sequences from ART-naïve and ART-experienced individuals using interpretable machine learning algorithms. Extreme Gradient Boosting (XGBoost), Gradient Boosting Machines (GBM), Random Forests (RF), Support Vector Machines (SVM), Logistic Regression (LR), and Naïve Bayes classifiers were trained to distinguish treatment status based solely on HIV-1 integrase mutation profiles. XGBoost outperformed other classifiers, with an AUROC of 0.92. Top-ranking mutations identified by the classifiers, including S283G, T112V, D278A, K136Q, T125A, V201I, V31I, I72V, A265V, G134N, and I135V among others, were significantly more prevalent in ART-experienced sequences. Structural analysis showed that most mutations potentially destabilize the three-dimensional structure of HIV-1 integrase. Relative risk (RR) analysis identified twenty three significant co-occurring mutation pairs with major INSTI resistance mutations, including G118R-R269K (RR = 2.3), Q148H-T125A (RR = 1.6), and Y143A-I135V (RR = 2.3). Most of these associations clustered within established resistance pathways (G118R, Q148/G140, Y143, and N155).

**Conclusions:**

Machine learning identified potential accessory resistance-associated mutations in HIV-1 integrase beyond established INSTI resistance pathways. These mutations may contribute to INSTI resistance via epistatic interactions with known major resistance mutations. Validation of the predictive framework using independent clinical datasets, together with experimental and longitudinal studies, is required to determine the functional impact of these mutations and their relevance to treatment outcomes.

**Supplementary Information:**

The online version contains supplementary material available at https://doi.org/10.1186/s12864-026-13243-3.

## Introduction

Integrase strand transfer inhibitors (INSTIs) prevent the replication of HIV by blocking integrase, a viral protein responsible for integrating viral DNA into the host cell genome [1, 2]. Several INSTIs have been approved by the United States Food and Drug Administration (FDA), including raltegravir (RAL), elvitegravir (EVG), dolutegravir (DTG), and bictegravir (BIC) and cabotegravir (CAB) [3]. The World Health Organization (WHO) recommends DTG for first and second-line antiretroviral therapy (ART) due to its high efficacy, favorable safety and high barrier to resistance, compared to non-nucleoside reverse transcriptase inhibitors (NNRTIs) [4].

Although data remain limited, there is emerging evidence of DTG resistance in non-B HIV-1 subtypes [5, 6] and cases of virological failure have been reported in absence of major drug resistance mutations [7, 8]. While several of these cases may be attributed to factors such as suboptimal adherence, these observations also underscore the importance of exploring alternative resistance mechanisms against contemporary ART regimens.

Conventional genotypic methods for identifying drug resistance mutations can be constrained in some settings by limited access to sequencing infrastructure, a shortage of technical expertise, and extended turnaround times, factors that vary considerably across resource-limited settings [9]. Genotyping involves sequencing the viral genome and identifying known drug resistance mutations, while phenotyping involves testing viral replication in the presence of different drugs to determine drug susceptibility [10–12].

Established computational resistance interpretation tools incorporate extensive experimental, clinical, and phenotypic data to capture complex resistance patterns. Machine learning approaches offer a complementary means of identifying previously uncharacterized mutation associations directly from large-scale sequence datasets to identify interactions that may contribute to alternative resistance pathways [9]. Machine learning algorithms have been used to identify patterns of drug resistance mutations and predict the likelihood of antiretroviral drug resistance based on mutation profiles [13–16]. A study that applied support vector machines on HIV-1 sequences from RAL-experienced and RAL-naïve individuals, identified two novel resistance mutation, I203M and I208L, associated with treatment failure [17]. A similar study that used random forests, logistic regression, and Naive Bayes algorithms to classify reverse transcriptase sequences between ART-experienced and ART-naïve individuals, identified six novel DRMs in reverse transcriptase i.e., L228R, L228H, E203K, D218E, I135L and H208Y [9].

In a similar approach, we applied interpretable machine learning algorithms to classify HIV-1 sequences from ART-experienced and ART-naive individuals, with an aim of identifying potentially novel resistance associated mutations in HIV-1 integrase and to assess the association of these mutations with known major INSTI resistance mutations.

## Methods

### Data acquisition and processing

We retrieved sequences from the Los Alamos HIV sequence database. The dataset was filtered to include sequences containing integrase sequences, with information on geographical region, date of sample collection and treatment status (i.e., ART-naïve, or ART-experienced). Sequences were uploaded to the Stanford University HIV drug resistance database algorithm (HIVdb) for identification of mutations and HIV drug resistance (HIVDR) interpretation [18]. HIVDR reports were combined with treatment status and subtype information, retaining integrase gene mutation profiles for further analysis. To prepare the data for machine learning, a binary matrix was created with sequence names in rows and mutations in columns. Mutations were represented using one-hot encoding, with each mutation represented as a binary vector of 0 s and 1 s to indicate the absence or presence of the mutation respectively. We excluded mixture mutations and two datasets were created: one including all mutations and another excluding known resistance associated mutations. For each dataset, we used Lasso regularization for feature selection to account for potential feature interactions. To account for the over-representation of subtype B relative to other subtypes and CRFs, we grouped samples into two categories, subtype B and non-B, and applied stratified sampling with respect to subtype group and the target variable before model training. For each dataset, we used stratified k-fold cross-validation to preserve class balance across folds. We tuned hyperparameters using grid search nested within each fold to prevent data leakage.

### Data description

We retrieved a total of 41,247 sequences, of which 19,762 (47.9%) were from ART-naïve and 21,485 (52.1%) were from ART-experienced individuals, with a sampling timeframe of 1996 to 2022. Geographically, most sequences were from North America 17,764 (43.1%) followed by Asia 8,495 (20.6%), Africa 7,665 (18.6%)**,** Europe 4,232 (10.3%), South America 441 (1.1%) and others 2,650 (6.4%).

The proportion of ART-experienced and ART-naïve sequences was not evenly distributed across regions. Most sequences from North America (83.1%) were from ART-naïve individuals. However, for Europe and South America, 42.5% and 60.1% of sequences, respectively, were from ART-experienced individuals. In addition, most sequences from Africa (89.1%), Asia (87.9%), and other regions (80.5%) were also from ART-experienced individuals.

**Fig. 1:**
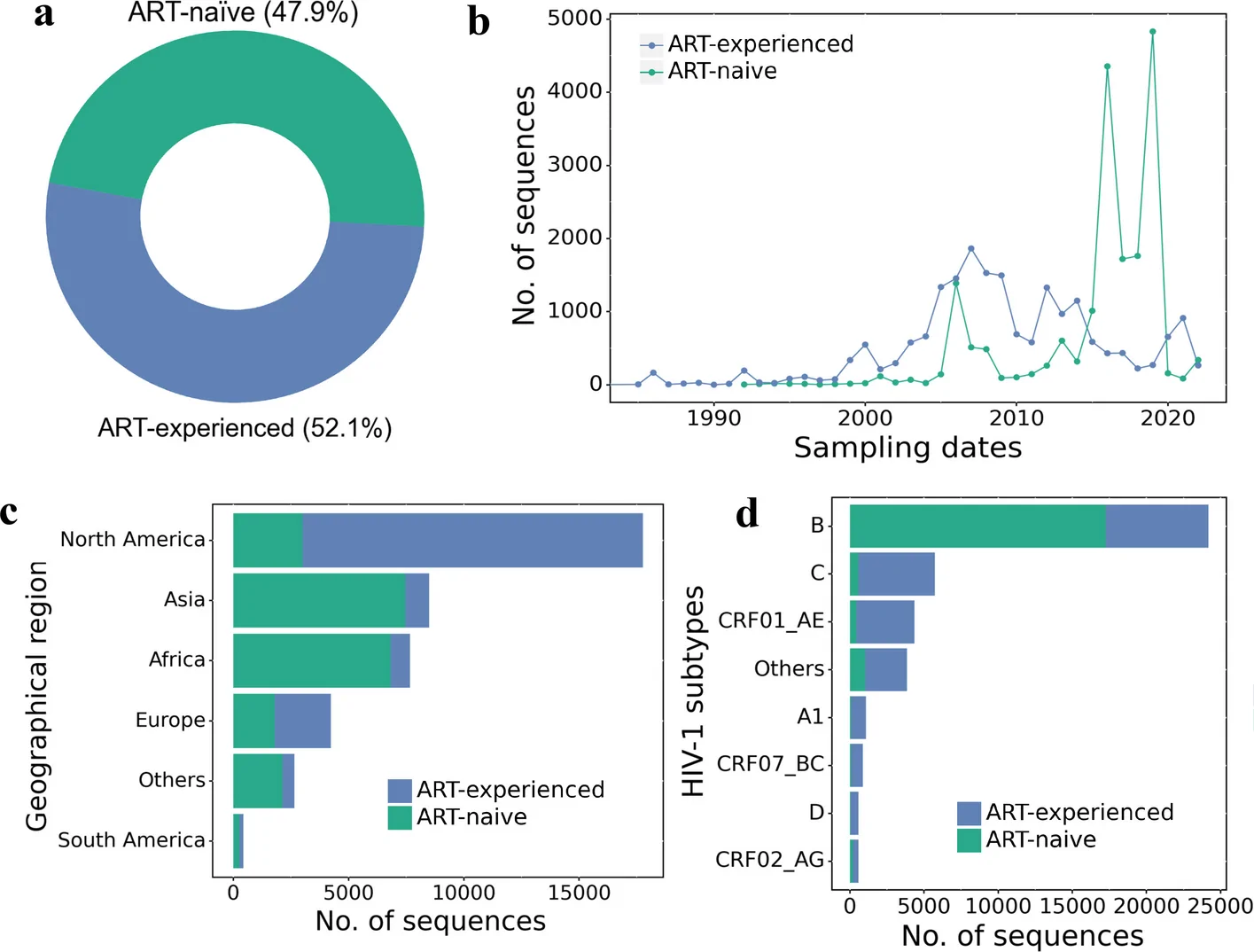
Overview of the dataset used in this study. **a** Proportion of sequences derived from antiretroviral therapy (ART)-experienced and ART-naïve individuals. **b** Temporal distribution of sequences included in the study, stratified by treatment status. The number of sequences per sampling year shows an overall increase over time. **c** Geographical distribution of sequences across continents. **d** Distribution of HIV-1 subtypes and recombinant forms in the dataset

Most sequences where subtype B, with 24,185 sequences (58.6%), followed by subtype C with 5,729 sequences (13.9%), CRF01_AE with 4,356 (10.6%), subtype A1 with 1,088 (2.6%), CRF07_BC, D and CRF02_AG with 872 (2.1%), 588 (1.4%) and 578 (1.4%) respectively. 3,851 (9.3%) sequences were classified as other subtypes and recombinant forms. Major drug resistance mutations were generally rare, with G140S, N155H, E138K and Q148R being the most frequent, with prevalence range of 2% to slightly over 4% of all the sequences (Fig. 1). Following the removal of mixed mutations, the feature set decreased from 8,118 to 5,977 mutations. LASSO-based feature selection further reduced the number of mutations to an average of 1,006 and 934 for datasets with and without known resistance associated mutations repectively, per cross-validation fold across models.

### Machine learning classifiers

We selected machine learning classifiers which have been applied in HIV drug resistance and viral load suppression prediction studies. These include Naïve Bayes (NB), Support Vector Machine (SVM), logistic regression (LR) and random forest (RF). In addition, we explored gradient boosting machines (GBM) and extreme gradient boosting (XGBoost) to investigate whether boosting-based approaches could improve predictive performance on the classification task of distinguishing HIV-1 sequences from ART-naïve and ART-experienced individuals based on their HIV-1 integrase mutation profiles. We used the scikit-learn [19] and the xgboost Python libraries to train and evaluate the classifiers.

LR is a supervised classification algorithm that models the probability of a binary outcome. It estimates the log-odds of class membership as a linear combination of input features and parameters learned using maximum likelihood estimation [20]. The model provides an interpretable baseline by quantifying the direction and magnitude of association between HIV-1 integrase mutations and ART status.

SVM is a supervised learning algorithm that constructs an optimal decision boundary by maximizing the margin between two classes. Using kernel functions which include linear, polynomial, and radial basis function, SVMs can model both linear and non-linear decision boundaries [21]. For this study, we used a linear SVM kernel to allow direct interpretation of contributions of individual mutation to the classification task.

Random Forest is an ensemble learning method that constructs multiple decision trees using bootstrapped subsets of the training data. At each split within a tree, a random subset of features is considered, which introduces diversity among trees. Final predictions are obtained by aggregating the outputs of all trees, taking majority voting for classification [22].

GBM is an ensemble method that builds decision trees sequentially, where each new tree is trained to correct the residual errors of the existing ensemble. The model minimizes a loss function using gradient descent [23].

### Performance metrics of machine learning classifiers

Classifier performance was evaluated using six metrics: accuracy, sensitivity, specificity, precision, F1-score, and area under the receiver operating characteristic curve (AUROC). Accuracy quantifies the overall proportion of correctly classified sequences among all sequences:

$$Accuracy= \frac{TP+TN}{TP+TN+FP+FN}$$

Sensitivity measures the proportion of sequences from ART experienced individuals that are correctly classified:

$$Sensitivity= \frac{TP}{TP+FN}$$

Specificity measures the proportion of sequences from ART-naïve individuals that are correctly classified:

$$Specificity= \frac{TN}{TN+FP}$$

Precision measures the proportion of sequences predicted to be ART experienced that are correctly classified.

$$Precision= \frac{TP}{TP+FP}$$

F1-score, defined as the harmonic mean of precision and sensitivity, provides a balance between the two metrics and measures overall classifier performance:

$$F1-score= \frac{2\times (Precision\times Sensitivity)}{Precision+Sensitivity}$$

The AUROC quantifies the overall ability of the classifier to distinguish sequences based on the ART status of the individuals from whom they were derived (ART-naïve and ART-experienced). It is area under the ROC curve, which is obtained by plotting the sensitivity (true positive rate) against the false positive rate (1-specificity).

In this study, TP, TN, FP, and FN denote number of true positives, true negatives, false positives, and false negatives, respectively. True positives are sequences from ART-experienced individuals correctly classified as such, while true negatives are sequences from ART-naïve individuals correctly identified. False positives correspond to sequences from ART-naïve individuals that are misclassified as ART-experienced, while false negatives are sequences from ART-experienced individuals misclassified as ART-naïve.

### Feature importance analysis

We used feature importance analysis to identify mutations that contributed strongly to distinguishing HIV sequences of ART-experienced individuals versus those of ART-naive individuals. For LR and SVM, we used the coefficients of each mutation to indicate the relative influence of each mutation. For RF and GBM, mutation importance was based on the mean decrease in impurity as a measure of how each mutation contributes to the classification task.

### Predicting changes in Gibbs free energy

We assessed the impact of the twenty most important mutations from the best performing classifier on stability of HIV-1 integrase using the mutation cut-off scanning matrix (mCSM) tool [24]. mCSM uses graph-based distance patterns of neighboring residues to estimate the impact of a mutation on protein stability by calculating the change in Gibbs free energy. Mutations are classified as stabilizing or destabilizing when the predicted change in Gibbs free energy is positive or negative, respectively [25].

### Further analysis of top ranked mutations

To identify potentially novel resistance-associated mutations (pRAMs), we extracted the twenty most important mutations from the best performing classifier. We then applied Fisher’s exact test to detect mutations that were significantly more prevalent in ART experienced individuals. To further assess their relevance, we calculated the relative risk (RR) to quantify the extent to which these mutations were overrepresented in sequences containing known major drug resistance mutations. The RR gives a measure of how much more frequently a given pRAM occurs in the presence of a specific major drug resistance mutation than in its absence. For each pRAM and each major drug resistance mutation (RAM), we constructed a 2 × 2 contingency table to evaluate their co-occurrence, as previously described [9].

**Table:** 

	RAM present	RAM absent
pRAM present	A	B
pRAM absent	C	D

The relative risk of the pRAM with respect to RAM is then defined as:

$$\mathrm{R}\mathrm{R} (\mathrm{p}\mathrm{R}\mathrm{A}\mathrm{M}, \mathrm{R}\mathrm{A}\mathrm{M})=\frac{A}{\left(A+C\right)}\div \frac{\mathrm{B}}{(B+D)}$$

where: A represents number of sequences with both the pRAM and RAM; B represents the number of sequences with the pRAM but without RAM; C represents the number of sequences without the pRAM but with RAM; D represents the number of sequences without either the pRAM or RAM. A value of RR *>* 1 indicates overrepresentation, while RR *<* 1 indicates underrepresentation of the pRAM in sequences containing a specific known major drug resistance mutation. DRM-pRAM pairs with RR ≥ 1.5, FDR-adjusted *p* < 0.05, and 95% confidence lower bound > 1.0 were used to construct a weighted bipartite network using the NetworkX library in Python.

## Results

### Classifier performance and interpretation

**Fig. 2:**
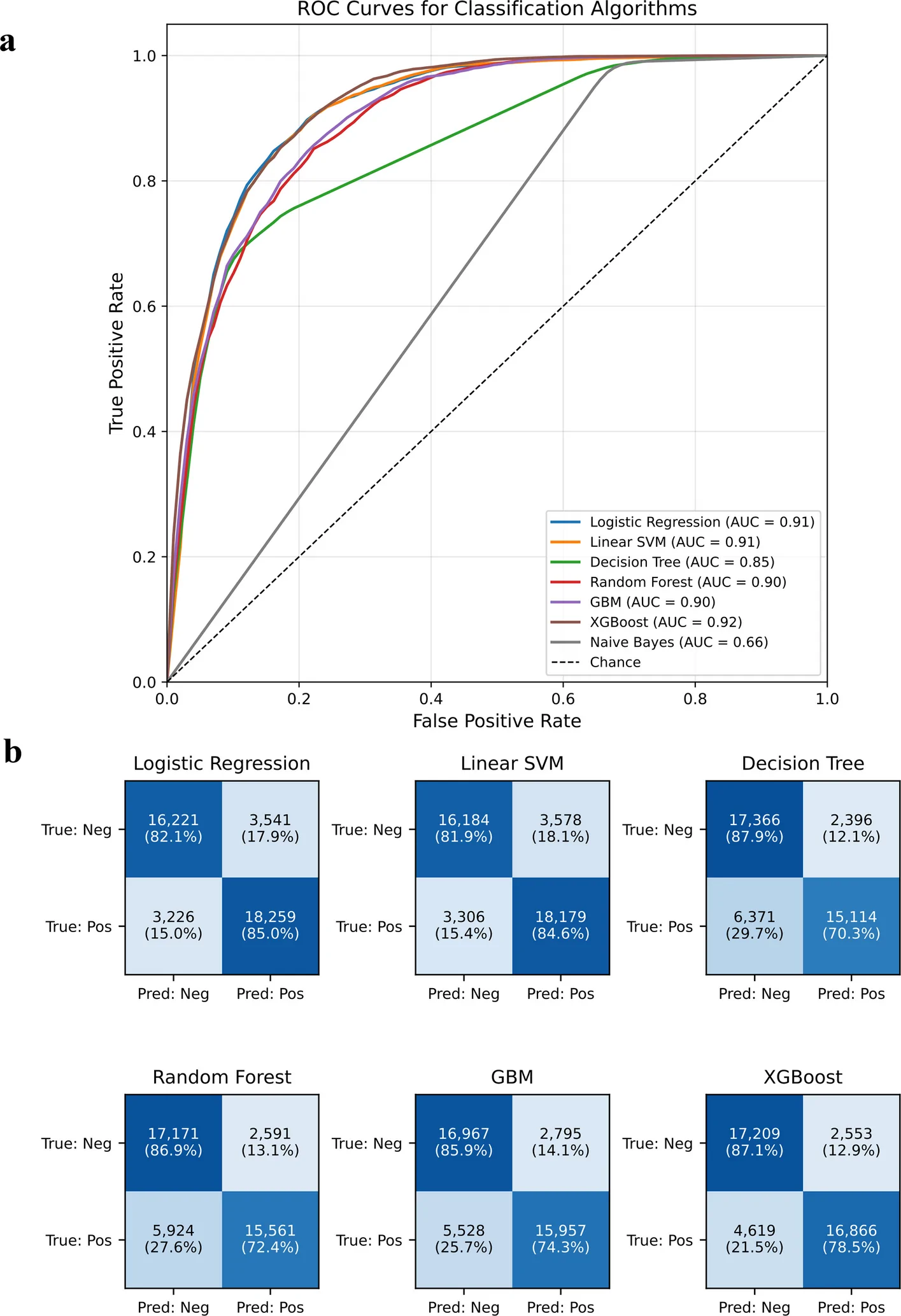
Performance evaluation of machine learning classifiers trained and tested on the dataset with known INSTI drug resistance mutations. **a** Receiver operating characteristic (ROC) curves. The area under the receiver operating characteristic curve (AUROC) shows overall classifier performance. **b** Confusion matrices for Gradient Boosting Machine (GBM), Logistic Regression (LR), Random Forest (RF), Decision Tree (DT), Extreme Gradient Boosting (XGBoost) and Linear SVM classifiers. Values represent the number of correctly and incorrectly classified sequences, with percentages indicating row-wise classification performance. Class labels correspond to treatment status: 0 denotes ART-naïve sequences, and 1 denotes ART-experienced sequences

The average performance metrics obtained over fivefold cross-validation for the seven classification algorithms evaluated on the dataset including known INSTI DRMs and the dataset excluding known INSTI DRMs are summarized in Table 1. Across both datasets, XGBoost showed the highest overall discriminative performance, with an AUROC of 0.92, followed by Logistic Regression and Linear SVM (AUROC = 0.90–0.91). Logistic Regression and Linear SVM consistently attained the highest accuracies (0.83–0.84) and F1-scores (0.84–0.85). The tree-based classifiers (XGBoost, Gradient Boosting, Random Forest, and Decision Tree) showed a pattern of higher specificity (0.86–0.88) than sensitivity (0.70–0.79), whereas Logistic Regression and Linear SVM showed higher sensitivities (0.85–0.87) with comparatively lower specificities (0.80–0.82). Naïve Bayes had the highest sensitivity (0.98–0.99) but the lowest specificity (0.27–0.33), resulting in the lowest overall performance (AUROC = 0.63–0.66). Similar performance trends were observed after excluding known INSTI resistance-associated mutations. Across all classifiers, the differences in performance metrics between the two datasets were minimal, with changes of utmost 3%. The receiver operating characteristic (ROC) curves and aggregated confusion matrices for the dataset containing known INSTI DRMs are presented in Fig. 2.

**Table 1:** Performance metrics of machine learning classifiers

		Accuracy	Sensitivity	Specificity	Precision	F1-score	AUROC
Dataset with known INSTI DRMs	XGB	0.83	0.79	0.87	0.87	0.83	0.92
	LR	0.84	0.87	0.81	0.83	0.85	0.91
	SVM	0.84	0.87	0.80	0.83	0.85	0.91
	GBM	0.80	0.75	0.86	0.85	0.80	0.90
	RF	0.80	0.74	0.87	0.86	0.79	0.90
	DT	0.78	0.72	0.87	0.86	0.78	0.85
	NB	0.67	0.99	0.33	0.61	0.76	0.66
Dataset without known INSTI DRMs	XGB	0.83	0.79	0.87	0.87	0.82	0.92
	LR	0.84	0.85	0.82	0.84	0.84	0.90
	SVM	0.83	0.85	0.82	0.84	0.84	0.90
	GBM	0.80	0.74	0.86	0.85	0.79	0.90
	RF	0.79	0.72	0.87	0.86	0.79	0.89
	DT	0.79	0.70	0.88	0.86	0.77	0.85
	NB	0.64	0.98	0.27	0.59	0.74	0.63

*AUROC* Area Under Receiver Operating Characteristic Curve, *SVM* Support Vector Machine, *LR* Logistic Regression, *GBM* Gradient Boosting Machine, *RF* Random Forest, *DT* Decision Trees, *NB* Naïve Bayes, *INSTI* Integrase Strand Transfer Inhibitors, *DRMs* Drug Resistance Mutations

### Feature importance to identify candidate resistance-associated mutations

To capture the complementary predictive signal reflected in the differing sensitivity–specificity trade-offs across model families, feature importance rankings were examined separately within each model family. We identified 50 top most ranked mutations were identified seperately for the tree-based models (decision tree, GBM, random forest, and XGBoost) and,, for the linear models (logistic regression and linear SVM), based on frequency of appearance and importance rank across models. Combining both lists yielded a set of 90 mutations among which 23 mutations were significantly more prevalence among sequences from ART-experienced individuals compared to ART-naïve individuals. Of these, sixteen (16) mutations which were identified in atleast 1,000 sequences (~ 2.4%) were retained for further analysis (Table 2).

**Table 2:** Prevalence of top-ranked mutations in ART-naive and ART-experienced individuals

Mutation	ART-naïve (*n* = 19,762)	ART-experienced (*n* = 21,485)	*P*-value
D278A	649 (3.3)	5650 (26.3)	< 0.0001
G134N	1,051 (5.3)	5667 (26.4)	< 0.0001
K136Q	1,237 (6.3)	6820 (31.7)	< 0.0001
S283G	2,673 (13.5)	12096 (56.3)	< 0.0001
T112V	2,167 (11.0)	11410 (53.1)	< 0.0001
T125A	5,973 (30.2)	13131 (61.1)	< 0.0001
V201I	9,358 (47.4)	15118 (70.4)	< 0.0001
I84M	398 (2.0)	2083 (9.7)	< 0.0001
S39N	471 (2.4)	2170 (10.1)	< 0.0001
A265V	1,758 (8.9)	4260 (19.8)	< 0.0001
I135V	2,745 (13.9)	5536 (25.8)	< 0.0001
R269K	1,605 (8.1)	3909 (18.2)	< 0.0001
V31I	6,032 (30.5)	9615 (44.8)	< 0.0001
I72V	6,186 (31.3)	8558 (39.8)	< 0.0001
G193E	888 (4.5)	1241 (5.8)	< 0.0001
K136T	527 (2.7)	657 (3.1)	0.01890

Numbers in brackets show the proportion of samples in which a particular mutation was identified. *P*-values were calculated using Chi-square test

### Distribution and co-occurrence of pRAMs with known resistance-associated mutations

All 16 pRAMs showed significantly different prevalence between subtype B and non-B sequences (Supplementary Table 1). With the exception of G193E, which was more prevalent in subtype B (5.85%) than non-B (4.19%), all other pRAMs were more prevalent in non-B than B sequences. The largest differences were observed for S283G (7.54% in B vs. 75.87% in non-B), T112V (2.69% vs. 75.76%), K136Q (0.48% vs. 46.54%), G134N (0.19% vs. 39.10%), and T125A (23.13% vs. 79.18%). Smaller but still significant differences were observed for I72V (27.96% vs. 46.78%), V31I (27.14% vs. 53.24%), and A265V (8.77% vs. 22.84%). K136T and R269K showed the lowest overall prevalence in subtype B (0.42% and 4.83%, respectively) relative to non-B subtypes (6.35% and 25.47%).

The prevalence of pRAMs varied signifcantly across geographic regions (Supplementary Table 2). Several mutations were higly prevalent in Africa, including A265V (48.58%), D278A (58.02%), K136Q (51.47%), and R269K (35.93%). Others were most prevalent in Asia, including G134N (52.20%), I135V (53.77%), T112V (72.30%), T125A (79.01%), V201I (82.57%), and V31I (54.60%). S283G showed comparably high prevalence in both Africa (74.95%) and Asia (73.15%), while I84M and S39N were most prevalent in Asia (14.95% and 23.01%, respectively) relative to other regions. I72V showed a relatively even distribution across Africa (42.22%), Asia (43.21%), and Others (41.17%), with lower prevalence in North America (28.95%). K136T and G193E were the least prevalent mutations overall, with K136T highest in Europe (8.08%) and G193E highest in the Others category (10.87%). Most mutations were least prevalent in South America.

**Fig. 3:**
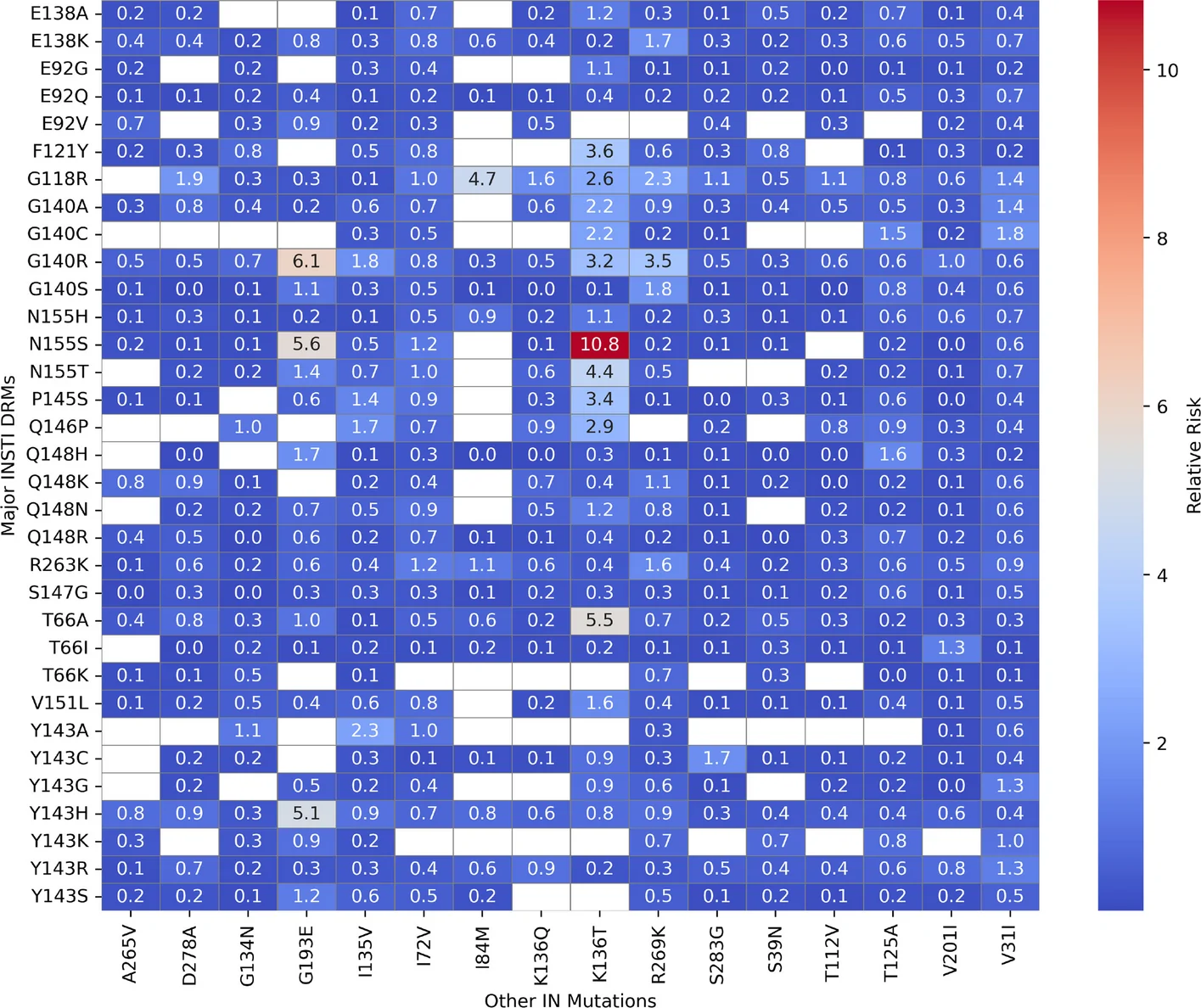
The heatmap shows pairwise relative risk values quantifying the strength of association between established integrase DRMs and other observed integrase (IN) mutations. Each cell represents the RR for a specific DRM-mutation pair. The color scale ranges from blue (lower relative risk) to red (higher relative risk). Higher relative risk values indicate increased co-occurrence

**Fig. 4:**
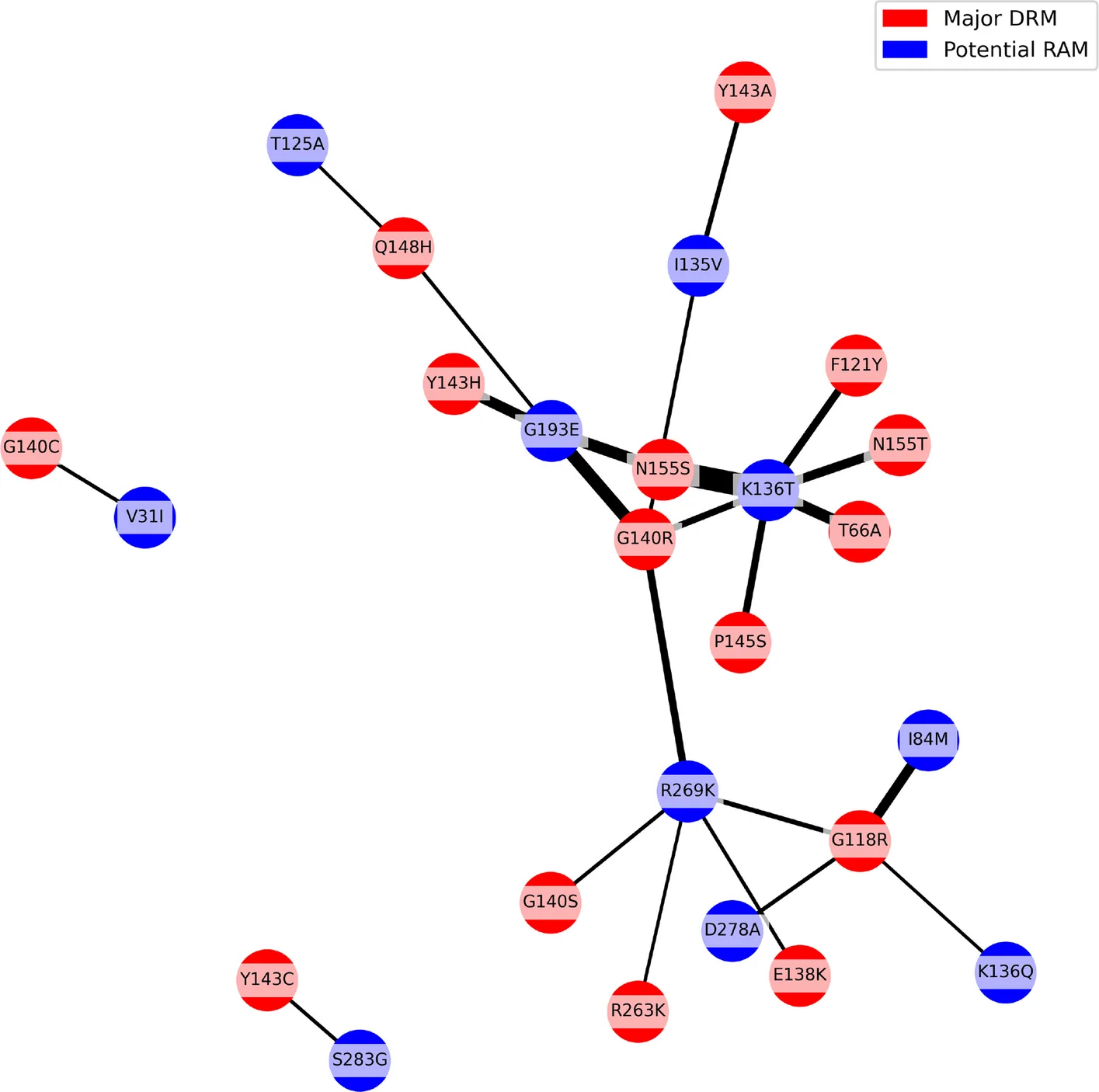
Network of statistically significant co-occurrence associations between major drug resistance mutations (DRMs) and potential resistance-associated mutations (pRAMs) in HIV integrase. Nodes represent individual mutations, classified as major DRMs (red) or pRAMs (purple). Edges represent statistically significant co-occurrence associations between DRM–pRAM pairs, identified using relative risk analysis and retained if they satisfy all three criteria: relative risk ≥ 1.5, FDR-adjusted *p* < 0.05, and a 95% confidence interval lower bound > 1.0. Disconnected clusters represent independent candidate resistance pathways

Relative risk analysis identified 23 significant DRM-pRAM pairs with (RR ≥ 1.5, FDR-adjusted *p* < 0.05, 95% CI lower bound > 1.0) after Benjamini–Hochberg correction (Table 3). K136T was the most frequently co-occurring pRAM, appearing alongside six major DRMs (F121Y, G140R, N155S, N155T, P145S, and T66A), with relative risk estimates ranging from 3.2 (G140R) to 10.8 (N155S) as shown in Fig. 3. R269K was the second most frequent, co-occurring with five major DRMs (E138K, G118R, G140R, G140S, and R263K; RR range 1.6–3.5), followed by G193E, which co-occurred with four major DRMs (G140R, N155S, Q148H, and Y143H; RR range 1.7–6.1). The strongest association overall was observed between N155S and K136T (RR = 10.8, 95% CI: 8.2–14.2, *p* < 0.0001), followed by G140R and G193E (RR = 6.1, 95% CI: 4.7–7.8, *p* < 0.0001) and T66A and K136T (RR = 5.5, 95% CI: 3.0–10.1, *p* < 0.0001). A network of these pRAM-DRM co-occurence pairs is shown in Fig. 4.

**Table 3:** Statistically significant co-occurrence associations between major drug resistance mutations and potential resistance-associated mutations

Major DRM	pRAM	a (DRM +/pRAM +)	b (DRM +/pRAM-)	c (DRM-/pRAM +)	d (DRM-/pRAM-)	RR	95 CI lower	95 CI upper	*P*-value	P-adjusted
E138K	R269K	118	393	5396	35340	1.743	1.485	2.046	< 0.0001	< 0.0001
F121Y	K136T	4	35	1180	40028	3.582	1.413	9.079	0.0251	0.038
G118R	D278A	20	48	6279	34900	1.929	1.334	2.79	0.0034	0.0061
G118R	I84M	19	49	2462	38717	4.673	3.184	6.859	< 0.0001	< 0.0001
G118R	K136Q	21	47	8036	33143	1.583	1.108	2.259	0.0304	0.0455
G118R	R269K	21	47	5493	35686	2.315	1.621	3.307	0.0002	0.0004
G140C	V31I	21	10	15626	25590	1.787	1.401	2.279	0.0012	0.0023
G140R	G193E	44	99	2085	39019	6.066	4.727	7.784	< 0.0001	< 0.0001
G140R	I135V	51	92	8230	32874	1.781	1.428	2.222	< 0.0001	< 0.0001
G140R	K136T	13	130	1171	39933	3.191	1.895	5.375	0.0003	0.0006
G140R	R269K	66	77	5448	35656	3.482	2.912	4.164	< 0.0001	< 0.0001
G140S	R269K	194	627	5320	35106	1.796	1.584	2.036	< 0.0001	< 0.0001
N155S	G193E	36	90	2093	39028	5.613	4.246	7.421	< 0.0001	< 0.0001
N155S	K136T	38	88	1146	39975	10.822	8.246	14.201	< 0.0001	< 0.0001
N155T	K136T	7	49	1177	40014	4.375	2.183	8.768	0.0011	0.0021
P145S	K136T	6	56	1178	40007	3.383	1.578	7.253	0.0087	0.0142
Q148H	G193E	33	346	2096	38772	1.698	1.222	2.358	0.0033	0.0059
Q148H	T125A	271	108	18833	22035	1.552	1.455	1.655	< 0.0001	< 0.0001
R263K	R269K	59	222	5455	35511	1.577	1.255	1.981	0.0004	0.0008
T66A	K136T	9	48	1175	40015	5.535	3.031	10.108	< 0.0001	< 0.0001
Y143A	I135V	10	12	8271	32954	2.266	1.433	3.582	0.0063	0.0106
Y143C	S283G	73	45	14696	26433	1.731	1.502	1.996	< 0.0001	< 0.0001
Y143H	G193E	11	31	2118	39087	5.095	3.062	8.48	< 0.0001	< 0.0001

Only pairs meeting all three criteria are shown: RR ≥ 1.5, FDR-adjusted *p* < 0.05, and 95% CI lower bound > 1.0

*DRM* drug resistance mutation, *RAM* resistance-associated mutation, *RR* relative risk, *CI* confidence interval

*P*-values were calculated using Fisher’s exact test and corrected for multiple comparisons using the Benjamini–Hochberg false discovery rate (FDR) method

^a^DRM present and RAM present

^b^DRM present and RAM absent

^c^DRM absent and RAM present

^d^DRM absent and RAM absent

### Predicted mutation-induced changes in Gibbs free energy

**Fig. 5:**
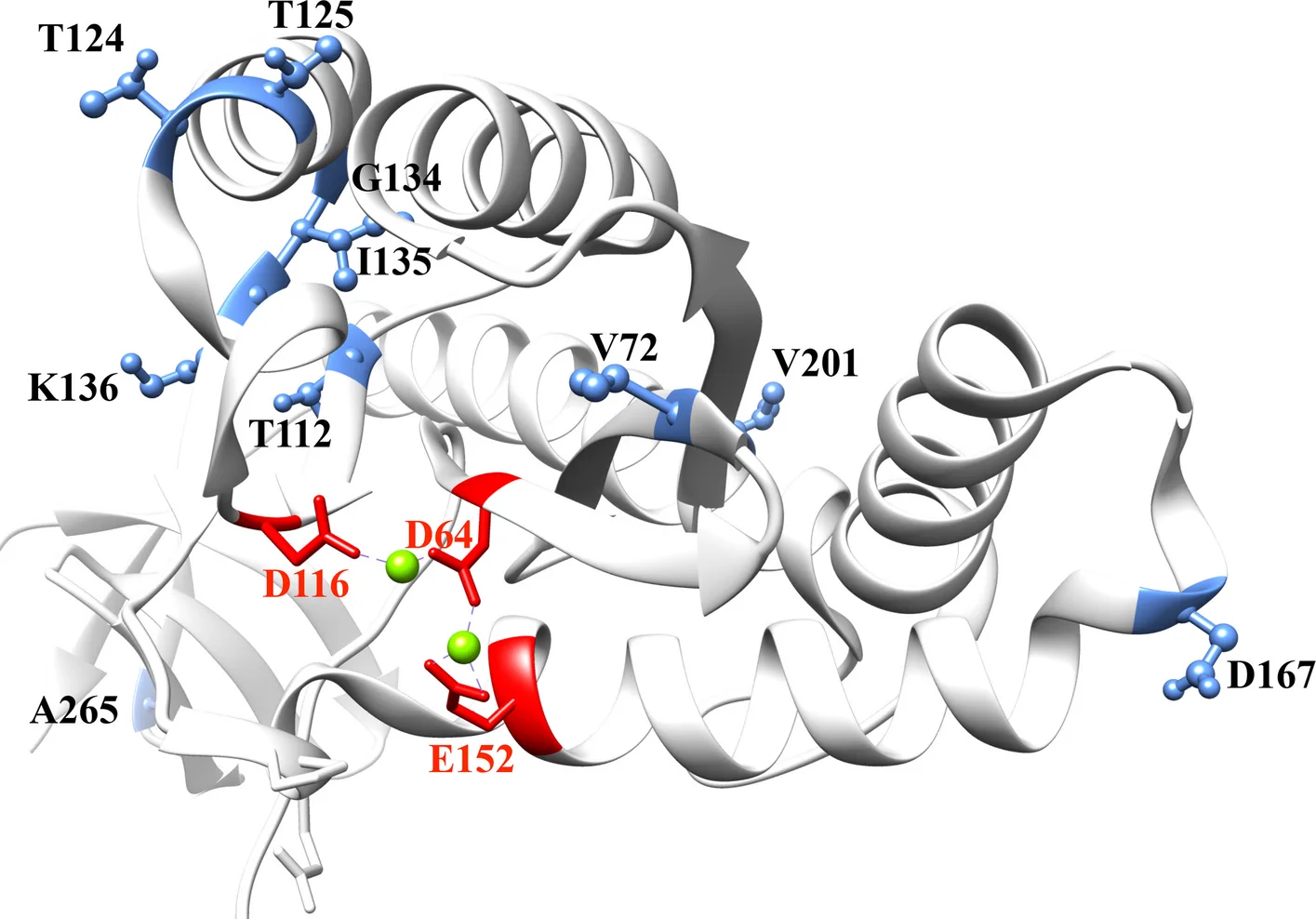
Structure of HIV-1 Integrase, chain A (PDB ID: 8W34.1) showing positions of identified mutations, that are in proximity with the catalytic triad. The residues are shown as stick and ball with color blue. Residues of the catalytic triad (D64, D116 and E152) are shown as sticks and colored red. Two magnesium ions that coordinate the binding of INSTIs at the catalytic triad of HIV-1 integrase are shown as green spheres

Stability predictions on the structure of HIV-1 subtype B integrase using the mCSM server showed negative ΔΔG values for all mutations (Table 4). The largest reduction in predicted stability was observed for I84M (ΔΔG = −1.557), followed by I135V (ΔΔG = −1.382), while K136Q showed the weakest destabilizing effect (ΔΔG = −0.092). Relative solvent accessibility (RSA) values ranged from 0.8% to 98.1%, indicating that the mutations occurred in both buried and solvent-exposed regions of the protein. Highly exposed residues included G134N (98.1%), V31I (75.8%), and K136Q/K136T (72.3%), whereas I84M (0.8%), A265V (0.9%), I135V (1.0%), and V201I (2.9%) were located in buried regions. Predictions could not be performed for mutations A265V, D278A, and S283G because the residues spanning positions 229–235 were unresolved in the 8W34.1 structure. In addition, the effect of I72V could not be evaluated, as position 72 in the 8W34.1 structure is already occupied by valine [26]. The three-dimensional structure of HIV-1 subtype B integrase (chain A), highlighting the positions of the identified pRAMs in proximity with the catalytic core domain, is shown in Fig. 5.

**Table 4:** Predicted effects of candidate HIV-1 integrase amino acid substitutions on protein structural stability

No	Mutation	RSA (%)	Predicted ΔΔG	Outcome
1	G134N	98.1	−0.61	Destabilizing
2	K136Q	72.3	−0.092	Destabilizing
3	T112V	22.9	−0.379	Destabilizing
4	T125A	40.1	−0.674	Destabilizing
5	V201I	2.9	−0.504	Destabilizing
6	I84M	0.8	−1.557	Destabilizing
7	S39N	57.2	−0.447	Destabilizing
8	A265V	0.9	−0.316	Destabilizing
9	I135V	1	−1.382	Destabilizing
10	V31I	75.8	−0.418	Destabilizing
11	G193E	45	−0.189	Destabilizing
12	K136T	72.3	−0.258	Destabilizing

Relative solvent accessibility (RSA, %) indicates the extent of residue surface exposure, while the predicted change in folding free energy (ΔΔG, kcal/mol) was used to assess the impact of each substitution on protein stability. Negative ΔΔG values indicate destabilizing effects, and all evaluated mutations were predicted to reduce integrase structural stability

## Discussion

In this study, we analyzed 41,247 HIV-1 integrase sequences using machine learning to identify mutational patterns associated with antiretroviral treatment exposure. The dataset included sequences representing diverse HIV-1 subtypes from six continents, with subtypes A1, B, C, D, CRF01_AE, CRF02_AG, and other circulating recombinant forms. Among the seven classification algorithms evaluated, XGBoost consistently showed the highest predictive performance, attaining an AUROC of 0.92 for both the complete dataset and the dataset excluding known integrase strand transfer inhibitor (INSTI) resistance-associated mutations. The superior performance of XGBoost shows that modeling complex, non-linear interactions among mutations provides additional predictive value beyond linear effects. The strong performance tree-based algorithms is consistent with previous studies where RF was superior to other algorithms in predicting resistance to reverse transcriptase and protease inhibitors [27, 28]. However, the comparable performance of Logistic Regression and Linear SVM (AUROC = 0.90–0.91) suggests that much of the predictive signal is captured by additive relationships between individual mutations and treatment exposure, with non-linear interactions contributing only a modest improvement.

The inclusion of known INSTI resistance-associated mutations had minimal impact on classifier performance, with differences of no more than 3% across all evaluation metrics. This shows that predictive information is not primarily driven by established resistance mutations such as G118R, Q148H, N155H, and Y143C, but is also captured by additional sequence variation within the integrase gene. The high performance across both datasets suggests that treatment-associated evolutionary signatures extend beyond resistance-associated mutations.

Feature importance rankings differed between tree-based and linear models, reflecting their differing sensitivity–specificity trade-offs. Several of these mutations showed marked differences in prevalence by subtype and geographic region, with most being substantially more common in non-B subtypes and concentrated in Africa or Asia.

Several top-ranking mutations identified including S283G, T112V, D278A, K136Q, T125A, V201I, V31I, I72V, A265V, G134N, and I135V were more prevalent among ART-experienced as compared to ART-naïve individuals. A number of these mutations have been previously reported in the context of INSTI treatment and HIVDR surveillance, though their direct contribution to resistance remains to be established [29–32]. Residues T112, K136, T125, V201, I72, G134 and I135 are part of the catalytic core domain, which harbors the active site for strand transfer activity [33, 34]. Structural analyses have shown that during strand transfer, residues T124 and T125 are in contact with the target DNA and variations at these sites may alter protein-DNA recognition [35]. Some mutations identified in the N-terminal domain of HIV-1 integrase have previously been implicated in modulating DNA binding and tetramer stability. V31I induces formation of a longer side chain that brings residue 31 within interacting distance of viral DNA, thereby influencing IN-DNA binding in non-B subtypes, whereas V31 does not directly interact with viral DNA. Other mutations in the NTD such as K14 contribute to tetramer stabilization through a salt bridge with W131, enabling formation of an additional salt bridge with W132, altering interaction energy [36].

Relative risk analysis identified several mutations with potential epistatic interactions with major INSTI resistance mutations. Twenty-three significant DRM-pRAM pairs were observed, with K136T showing the broadest co-occurrence, appearing alongside six major DRMs (F121Y, G140R, N155S, N155T, P145S, and T66A; RR range 3.2–10.8), followed by R269K, which co-occurred with five major DRMs (E138K, G118R, G140R, G140S, and R263K; RR range 1.6–3.5), and G193E, which co-occurred with four major DRMs (G140R, N155S, Q148H, and Y143H; RR range 1.7–6.1). The strongest associations overall were observed between N155S and K136T (RR = 10.8), G140R and G193E (RR = 6.1), and T66A and K136T (RR = 5.5). These findings show that potential resistance-associated mutations cluster within established INSTI resistance pathways. These polymorphisms may act as accessory mutations that increase resistance in combination with major INSTI resistance mutations, while others may serve a compensatory role by reducing the fitness burden associated with major resistance mutations [37].

Despite the robust machine learning framework applied in this study, several limitations should be considered. First, the models were trained on retrospective sequence data, where treatment exposure was used as the classification outcome rather than direct longitudinal measurements of INSTI treatment response or virological failure. Therefore, the identified mutation patterns represent associations with treatment history and may include signatures related to ART exposure, viral evolution, or subtype-specific background variation rather than mutations directly contributing to resistance. Future studies integrating longitudinal clinical data, including specific ART regimens, duration of treatment, and viral load, will be important to validate the clinical relevance of these predictions. Second, although feature selection and cross-validation strategies were implemented to reduce overfitting and data leakage, external validation using independent datasets from underrepresented populations and contemporary cohorts is required. Additionally, future studies could explore machine learning approaches that integrate sequence embeddings, protein structural information, evolutionary conservation scores, and interpretable deep learning methods to better capture complex epistatic interactions and identify biologically meaningful resistance-associated patterns. Finally, the predicted pRAMs and their structural effects require experimental validation through phenotypic susceptibility assays and functional studies to determine whether these mutations directly influence integrase inhibitor activity, viral fitness, or compensate for resistance-associated mutations.

## Conclusion

Using interpretable machine learning, we identified potential accessory resistance-associated mutations in HIV-1 integrase that are significantly overrepresented in sequences with known major INSTI drug resistance mutations. These mutations have been reported in previous studies. However, their role in drug resistance has not been experimentally confirmed. To fully understand their impact, there is need for longitudinal studies to investigate their association with treatment outcomes and functional studies to explore their role in development of resistance to contemporary ART regimens.

## Supplementary Information

Additional file 1. List of HIV-1 sequence identifiers in the Los Alamos HIV sequence database, corresponding GenBank accession numbers, and the treatment status of the individuals at the time of sample collection (yes denotes treatment-experienced; no denotes treatment-naïve); Fold-wise performance metrics and feature importance of all models.

Additional file 2: Supplementary Tables 1 and 2.

## Data Availability

The sequence data analyzed during the current study are available in the Los Alamos National Laboratory (LANL) HIV database. The GenBank accession numbers of these sequences are included in the supplementary information [see Additional file 1]. Analysis scripts are available and accessible on GitHub: https://github.com/AlfredUg/ML4HIVDR.

## Abbreviations

HIV: Human immunodeficiency virus
ART: Antiretroviral therapy
IN: Integrase
INSTI: Integrase strand transfer inhibitor
DTG: Dolutegravir
DRM: Drug resistance mutation
RAM: Resistance-associated mutation
HIVDR: HIV drug resistance
RF: Random forests
SVM: Support vector machines
GBM: Gradient boosting machines
LR: Logistic regression
ROC: Receiver operating characteristic
AUROC: Area under the receiver operating characteristic curve
RR: Relative risk
FDR: False discovery rate
mCSM: Mutation cut-off scanning matrix
pRAM: Potentially novel resistance-associated mutation
CRF: Circulating recombinant form
