# Supplementary material for: Machine learning identifies potential accessory resistance-associated mutations in HIV-1 integrase
Source: BMC Genomics. 2026 Aug 3;27:893. doi: 10.1186/s12864-026-13243-3 (PMC13721372; doi:10.1186/s12864-026-13243-3)
Supplement: Supplementary file 1 [file 12864_2026_13243_MOESM2_ESM.docx]

**Supplementary Table 1.** Prevalence of candidate pRAMs by HIV-1 subtype (B vs. non-B). Values represent n (%) of sequences carrying each mutation within the respective subtype group. P-values were derived from chi-square or Fisher's exact tests, as appropriate, with Benjamini-Hochberg correction for multiple comparisons.

| **Mutation** | **B (n=24,185)** | **non-B (n=17,062)** | **P-value** |
| --- | --- | --- | --- |
| A265V | 2121 (8.77%) | 3897 (22.84%) | < 0.0001 |
| D278A | 94 (0.39%) | 6205 (36.37%) | < 0.0001 |
| G134N | 46 (0.19%) | 6672 (39.1%) | < 0.0001 |
| I135V | 2697 (11.15%) | 5584 (32.73%) | < 0.0001 |
| I72V | 6763 (27.96%) | 7981 (46.78%) | < 0.0001 |
| I84M | 317 (1.31%) | 2164 (12.68%) | < 0.0001 |
| K136Q | 116 (0.48%) | 7941 (46.54%) | < 0.0001 |
| R269K | 1168 (4.83%) | 4346 (25.47%) | < 0.0001 |
| S283G | 1824 (7.54%) | 12945 (75.87%) | < 0.0001 |
| S39N | 422 (1.74%) | 2219 (13.01%) | < 0.0001 |
| T112V | 651 (2.69%) | 12926 (75.76%) | < 0.0001 |
| T125A | 5595 (23.13%) | 13509 (79.18%) | < 0.0001 |
| V201I | 10120 (41.84%) | 14356 (84.14%) | < 0.0001 |
| V31I | 6564 (27.14%) | 9083 (53.24%) | < 0.0001 |
| K136T | 101 (0.42%) | 1083 (6.35%) | < 0.0001 |
| G193E | 1414 (5.85%) | 715 (4.19%) | < 0.0001 |

**Supplementary Table 2.** Prevalence of candidate pRAMs by geographic region. Values represent n (%) of sequences carrying each mutation within the respective region.

| **Mutation** | **Africa (n=7,665)** | **Asia (n=8,495)** | **Europe (4,232)** | **North America (n=17,764)** | **Others (n=2,650)** | **South America (441)** |
| --- | --- | --- | --- | --- | --- | --- |
| A265V | 3724 (48.58%) | 171 (2.01%) | 220 (5.2%) | 1747 (9.83%) | 56 (2.11%) | 100 (22.68%) |
| D278A | 4447 (58.02%) | 1542 (18.15%) | 94 (2.22%) | 102 (0.57%) | 28 (1.06%) | 86 (19.5%) |
| G134N | 1003 (13.09%) | 4434 (52.2%) | 392 (9.26%) | 107 (0.6%) | 782 (29.51%) | 0 (0.0%) |
| I135V | 676 (8.82%) | 4568 (53.77%) | 447 (10.56%) | 2166 (12.19%) | 407 (15.36%) | 17 (3.85%) |
| I84M | 914 (11.92%) | 1270 (14.95%) | 47 (1.11%) | 138 (0.78%) | 110 (4.15%) | 2 (0.45%) |
| K136Q | 3945 (51.47%) | 2401 (28.26%) | 344 (8.13%) | 161 (0.91%) | 1056 (39.85%) | 150 (34.01%) |
| R269K | 2754 (35.93%) | 1276 (15.02%) | 307 (7.25%) | 984 (5.54%) | 101 (3.81%) | 92 (20.86%) |
| S283G | 5745 (74.95%) | 6214 (73.15%) | 951 (22.47%) | 1219 (6.86%) | 517 (19.51%) | 123 (27.89%) |
| S39N | 91 (1.19%) | 1955 (23.01%) | 59 (1.39%) | 383 (2.16%) | 147 (5.55%) | 6 (1.36%) |
| T112V | 4917 (64.15%) | 6142 (72.3%) | 750 (17.72%) | 371 (2.09%) | 1311 (49.47%) | 86 (19.5%) |
| T125A | 5138 (67.03%) | 6712 (79.01%) | 1732 (40.93%) | 3934 (22.15%) | 1399 (52.79%) | 189 (42.86%) |
| V201I | 5609 (73.18%) | 7014 (82.57%) | 1867 (44.12%) | 7858 (44.24%) | 1904 (71.85%) | 224 (50.79%) |
| V31I | 3356 (43.78%) | 4638 (54.6%) | 1336 (31.57%) | 4679 (26.34%) | 1492 (56.3%) | 146 (33.11%) |
| K136T | 446 (5.82%) | 95 (1.12%) | 342 (8.08%) | 160 (0.9%) | 140 (5.28%) | 1 (0.23%) |
| I72V | 3236 (42.22%) | 3671 (43.21%) | 1444 (34.12%) | 5143 (28.95%) | 1091 (41.17%) | 159 (36.05%) |
| G193E | 144 (1.88%) | 498 (5.86%) | 297 (7.02%) | 888 (5.0%) | 288 (10.87%) | 14 (3.17%) |
